# Correction: Human Papillomavirus Type 6 and 11 Genetic Variants Found in 71 Oral and Anogenital Epithelial Samples from Australia

**DOI:** 10.1371/journal.pone.0117962

**Published:** 2015-02-06

**Authors:** The PLOS ONE Staff

## Notice of Republication

This article was republished on January 2, 2015, to include the Supporting Information files, which were mistakenly left out of the original publication. Please download this article again to view the correct files. The originally published, uncorrected article and the republished, corrected article are provided here for reference.

## Supporting Information

S1 File Originally published, uncorrected article. (PDF)

S2 File Republished, corrected article. (PDF)
