# Supplementary material for: Human Papillomavirus Type 6 and 11 Genetic Variants Found in 71 Oral and Anogenital Epithelial Samples from Australia
Source: PLoS One. 2013 May 17;8(5):e63892. doi: 10.1371/journal.pone.0063892 (PMC3656832; doi:10.1371/journal.pone.0063892)
Supplement: Table S1 — Summary of HPV6 and HPV11 lesion types. (DOCX) [file pone.0063892.s001.docx]

**Table S1.** Summary of HPV6 and HPV11 lesion types.

|  | **Genital Warts** | **Cervical Cells** | **RRP** | **Anal Cancer** | **Total** |
| --- | --- | --- | --- | --- | --- |
| **HPV6** | 33 | 4 | 8 | 4 | 49 |
| **HPV11** | 10 | 1 | 9 | 2 | 22 |
| **Total** | 43 | 5 | 17 | 6 | 71 |

**Two tailed P value of HPV 6 associated with anogenital lesion: 0.036**
