# Supplementary material for: Human Papillomavirus Type 6 and 11 Genetic Variants Found in 71 Oral and Anogenital Epithelial Samples from Australia
Source: PLoS One. 2013 May 17;8(5):e63892. doi: 10.1371/journal.pone.0063892 (PMC3656832; doi:10.1371/journal.pone.0063892)
Supplement: Table S2 — Summary of genetic variation identified across all lesion types for HPV6 and HPV11. (DOCX) [file pone.0063892.s002.docx]

**Table S2.** Summary of genetic variation identified across all lesion types for HPV6 and HPV11.

|  | **E6** | **E7** | **LCR** |
| --- | --- | --- | --- |
| **HPV6** |  |  |  |
| Total single nucleotide alterations | 13 | 5 | 45 |
| Total insertions / deletions |  |  | 5 / 4 |
| Total amino acid alterations | 3 | 3 |  |
| **HPV11** |  |  |  |
| Total single nucleotide alterations | 6 | 2 | 13 |
| Total insertions / deletions |  |  | 1 / 3 |
| Total amino acid alterations | 3 | 2 |  |
| Total number of bases sequenced for HPV 6 | 453* | 297* | 853 |
| Total number of bases sequenced for HPV 11 | 453* | 297* | 760 |

***E6 and E7 have a 25 bp overlap.**
