# Supplementary material for: Human Papillomavirus Type 6 and 11 Genetic Variants Found in 71 Oral and Anogenital Epithelial Samples from Australia
Source: PLoS One. 2013 May 17;8(5):e63892. doi: 10.1371/journal.pone.0063892 (PMC3656832; doi:10.1371/journal.pone.0063892)
Supplement: Table S3 — Primers used for amplifying and sequencing the LCR, E6 and E7 ORFs. (DOCX) [file pone.0063892.s003.docx]

**Table S3**. Primers used for amplifying and sequencing the LCR, E6 and E7 ORFs.

|  |  | **Primer** | **Sequence (5’-3’)** | **Nucleotide Position** | **Amplicon Length** |
| --- | --- | --- | --- | --- | --- |
| **HPV 6** |  |  |  |  |  |
|  | **E6** | E6-1 | ATA GGA GGG ACC GAA AAC G | 26-44 | 245 bp |
|  |  | E6-1 | CTA AAG GTC CTG TTT CGA GG | 252-271 |  |
|  |  | E6-2 | GCA AGA ATG CAC TGA CCA C | 202-220 | 236 bp |
|  |  | E6-2 | TGT CAC AAA CCG CTG TGT G | 420-438 |  |
|  |  | E6-3 | GAC GTG CTA ATT CGG TGC TA | 396-415 | 240 bp |
|  |  | E6-3 | AGT AGA CAG CTC AGA AGA TGA | 616-636 |  |
|  | **LCR** | LCR-1 | CTG CTG CCC CTA AAC GTA A | 7251-7269 |  |
|  |  | LCR-1 | GTG TAA TGT GTA TGT GTG TTT ATG TGC |  |  |
|  |  | LCR-2 | ATA TGT GTG TGT GTG TTC TGT GTG T | 7435-7459 |  |
|  |  | LCR-2 | CCA CCA ATT TGT TAC AAC GTG TT |  |  |
|  |  | LCR-3 LONG | TTT TAT ATT TGC AAC CGT TTT CG | 7629-7654 | 412 bp |
|  |  | LCR-3 LONG | AAA TAG GAG GGA CCG AAA ACG | 24-44 |  |
|  |  | LCR-3 SHORT | GGT GCG GTA TTG CCT TAC TC | 7793-7812 | 248 bp |
|  |  | LCR-3 SHORT | CCT GTC TTT GTG TTA TAC TTT TAT GCA | 7818-7844 | 216 bp |
|  |  | LCR | CAC ACC CTA CAT ATT TCC TTC | 7971-7991 | 184 bp |
|  |  | LCR | ATA GAC CAG TTG TGC AAG AC | 138-157 |  |
|  |  |  |  |  |  |
| **HPV 11** | **E6** | E6-1 | GAG GAG GGA CCG AAA ACG | 27-44 | 244 bp |
|  |  | E6-1 | CTA AAG GTT GTG TGG CGA GA | 252-271 |  |
|  |  | E6-2 | CAG GAA TGC ACT GAC CAC C | 203-221 | 231 bp |
|  |  | E6-2 | CTG TGT CAC AAG CCG TTG | 417-434 |  |
|  |  | E6-3 | ATT CGT TGT TAC CTG TGT CAC | 405-425 | 231 bp |
|  |  | E6-3 | AGA AGA CAG CTC AGA AGA TGA | 616-636 |  |
|  | **LCR** | LCR-1 | CGA AAA CGT ACC AAA ACC A | 7250 - 7268 | 241 bp |
|  |  | LCR-1 | ATT ATG TGT GTC CTG TTA CAC CC | 7468 - 7490 |  |
|  |  | LCR-2 | GGA ATG TGT ATG TAT GTT TTT GTG C | 7432 - 7456 | 240 bp |
|  |  | LCR-2 | CTA ATC CCA TAT GTT GTG TGC C | 7650 - 7671 |  |
|  |  | LCR-3 LONG | TTC GGT TGC CCT TAC ATA CAC T | 7598 - 7619 | 378 bp |
|  |  | LCR-3 LONG | AAA GAG GAG GGA CCG AAA ACG | 24 - 44 |  |
|  |  | LCR-3 SHORT | CGG TTT GTA CAA TGT TGT GGA T | 7759 - 7780 | 217 bp |
|  |  | LCR-3 SHORT | GAT TGC AGC CAA AGG TTA AAA G | 7778 - 7799 | 202 bp |
|  |  | LCR | CAC ACC CTA CAT ATT TCC TTC | 7906-7926 | 165 bp |
|  |  | LCR | ATA GAC CAG TTG TGC AAG AC | 138-157 |  |
| **HPV6/11** | **E7** |  |  |  |  |
|  |  | E7 | TAG GGT TAC ATT GCT ATG AGC | 591-611 | 263 bp |
|  |  | E7 | CGG ACG ATT CAG GTA CAG A | 836-854 |  |
