# Supplementary material for: Human Papillomavirus Type 6 and 11 Genetic Variants Found in 71 Oral and Anogenital Epithelial Samples from Australia
Source: PLoS One. 2013 May 17;8(5):e63892. doi: 10.1371/journal.pone.0063892 (PMC3656832; doi:10.1371/journal.pone.0063892)
Supplement: Table S4 — HPV6 nucleotide and amino acid sequence variation in the ORF of E6 and E7 from 49 clinical isolates representing four different lesion types. (DOCX) [file pone.0063892.s004.docx]

**Table S4**. HPV6 nucleotide and amino acid sequence variation in the ORF of E6 and E7 from 49 clinical isolates representing four different lesion types.

|  |  |  | **HPV6 E6/E7 Variant Groups** | | | | | | | | | | | |  |  |
| --- | --- | --- | --- | --- | --- | --- | --- | --- | --- | --- | --- | --- | --- | --- | --- | --- |
| **Nucleotide Position** | **ORF** | **Ref** X00203 | **A-1** | **A-2** | **B3-1** | **B3-2** | **B3-3** | **B3-4** | **B1** | **B1-1** | **B1-2** | **B1-3** | **B1-4** | **B1-5** | **Amino Acid** | **Frequency** |
| 156 | **E6** | **A** |  |  |  | T | T | T |  |  |  |  |  |  | E19K | 10 |
| 221 | **E6** | **A** | T | T | T | T | T | T | T | T | T | T | T | T |  | 49 |
| 251 | **E6** | **C** |  |  | G | G | G | G | G | G | G | G | G | G | H50Q | 44 |
| 311 | **E6** | **T** |  |  |  |  |  |  |  |  |  |  | C |  |  | 1 |
| 323 | **E6** | **A** |  |  | C | C | C | C | T | T | T | T | T | T |  | 12(C)/32(T) |
| 350 | **E6** | **T** |  |  |  | G |  |  |  |  |  |  |  |  |  | 3 |
| 365 | **E6** | **A** |  |  | T | T | T | T | T | T | T | T | T | T |  | 44 |
| 380 | **E6** | **T** |  |  |  |  |  |  |  | G |  |  |  |  |  | 1 |
| 392 | **E6** | **C** |  |  | T | T | T | T | T | T | T | T | T | T |  | 44 |
| 398 | **E6** | **C** |  |  |  |  |  |  |  |  | T |  |  |  |  | 1 |
| 440 | **E6** | **A** |  |  |  |  |  |  |  |  |  | C |  |  | E113D | 1 |
| 473 | **E6** | **G** | A | A | A | A | A | A | A | A | A | A | A | A |  |  |
| 479 | **E6** | **C** |  |  | T |  |  |  |  |  |  |  |  |  |  | 2 |
| 684 | **E7** | **T** |  |  |  |  |  |  | A | A | A | A | A | A | F52Y | 32 |
| 746 | **E7** | **G** |  | A |  |  |  |  |  |  |  |  |  |  | E73K | 1 |
| 791 | **E7** | **A** |  |  | G |  |  |  |  |  |  |  |  |  | N88D | 2 |
| 820 | **E7** | **G** |  |  |  |  | A |  |  |  |  |  |  | A |  | 3 |
| 823 | **E7** | **C** |  |  | A | A | A | A | A | A | A | A | A | A |  | 44 |
| **Lesion Type** | | |  |  |  |  |  |  |  |  |  |  |  |  |  |  |
| Anal cancer | | |  | 1 |  |  |  | 1 | 2 |  |  |  |  |  |  |  |
| Cervical Cells | | |  |  |  |  |  |  | 3 |  |  |  | 1 |  |  |  |
| Genital Warts | | | 1 |  | 2 | 2 | 1 | 2 | 20 | 1 | 1 | 1 |  | 2 |  |  |
| Recurrent Respiratory Papillomatosis | | | 3 |  |  | 1 |  | 3 | 1 |  |  |  |  |  |  |  |
| **Total** | | | **4** | **1** | **2** | **3** | **1** | **6** | **26** | **1** | **1** | **1** | **1** | **2** |  |  |
| **P Values for association with anogenital lestion** | | |  |  |  |  |  | **0.06** | **0.02** |  |  |  |  |  |  |  |

Nucleotide positions given are from the reference sequence HPV 6b (GenBank Acc. No X00203). Variant groups are assigned by HPV6 lineage A, and lineage B which is denoted according to sublineages B3 and B1. The frequency indicates the number of isolates for each variant identified across all HPV6 variant groups. Two-tailed P values were calculated using Fisher exact test.
