# Supplementary material for: Human Papillomavirus Type 6 and 11 Genetic Variants Found in 71 Oral and Anogenital Epithelial Samples from Australia
Source: PLoS One. 2013 May 17;8(5):e63892. doi: 10.1371/journal.pone.0063892 (PMC3656832; doi:10.1371/journal.pone.0063892)
Supplement: Table S5 — HPV6 nucleotide sequence variation in the LCR from 48 clinical isolates representing four different lesion types. (DOCX) [file pone.0063892.s005.docx]

**Table S5**. HPV6 nucleotide sequence variation in the LCR from 48 clinical isolates representing four different lesion types.

|  |  | | **HPV 6 LCR Variant Groups** | | | | | | | | | | | | | | | | | | | | | | | | | | | | | | | | | |  |
| --- | --- | --- | --- | --- | --- | --- | --- | --- | --- | --- | --- | --- | --- | --- | --- | --- | --- | --- | --- | --- | --- | --- | --- | --- | --- | --- | --- | --- | --- | --- | --- | --- | --- | --- | --- | --- | --- |
| **Nucleotide Position** | **Ref** X00203 | | **A1** | **A 2** | **A3** | **B3**  **1** | | **B3**  **2** | | | **B1**  **1** | **B1**  **2** | | **B1**  **3** | **B1**  **4** | **B1**  **5** | **B1**  **6** | **B1**  **7** | **B1**  **8** | | **B1**  **9** | **B110** | **B1 11** | **B1 12** | **B1 13** | **B1**  **14** | **B1 15** | **B1 16** | | **B1 17** | **B1**  **18** | **B1 19** | **B120** | **B121** | | **B122** | **Freq** |
| 7332 | **C** | |  |  |  |  | |  | | |  |  | |  |  |  |  |  |  | |  |  |  |  | G |  |  |  | |  |  |  |  |  | |  | 1 |
| 7337 | **A** | |  |  |  |  | |  | | |  |  | |  |  |  |  |  |  | | G |  |  |  |  |  |  |  | |  |  |  |  |  | |  | 1 |
| 7349 | **G** | |  |  |  |  | |  | | | T | T | | T | T | T | T | T | T | | T | T | T | T | T | T | T | T | | T | T | T | T | T | | T | 41 |
| 7356 | **C** | |  |  |  |  | |  | | |  | G | |  | G |  |  |  |  | |  |  |  |  |  |  |  |  | |  |  |  |  |  | |  | 4 |
| 7365/7385 |  | |  |  |  |  | |  | | | * |  | |  |  |  |  |  |  | |  |  |  |  |  |  |  |  | |  |  |  |  |  | |  |  |
| 7398 | **G** | |  |  |  |  | |  | | |  |  | |  |  |  |  |  |  | |  |  |  |  |  |  |  |  | |  |  | A |  |  | |  | 1 |
| 7400 | **C** | |  | A | A | A | | A | | | A | A | | A | A | A | A | A | A | | A | A | A | A | A | A | A | A | | A | A | A | A | A | | A | 47 |
| 7422 | **G** | |  |  |  |  | |  | | |  |  | |  |  |  |  |  |  | |  |  |  |  |  |  |  |  | |  |  | A |  |  | |  | 1 |
| 7452 | **C** | |  |  |  | A | | A | | | A | A | | A | A | A | A | A | A | |  |  |  | A | A | A | A | A | |  | A | A |  |  | |  | 35 |
| 7467 | **G** | | T | T | T | T | | T | | | T | T | | T | T | T | T | T | T | | T | T | T | T | T | T | T | T | | T | T | T | T | T | | T | 48 |
| 7513 | **C** | |  |  |  | A | | A | | | A | A | | A | A | A | A | A |  | | A | A | A | A | A | A | A | A | |  |  | A | A | A | | A | 40 |
| 7514/7515 |  | |  |  |  |  | |  | | | + 14 | + 14 | | + 14 | + 14 | + 14 | + 14 | + 14 | + 14 | | + 14 | + 14 | + 14 | + 14 | + 14 | + 14 | + 14 | + 14 | | + 14 | + 14 | + 14 | + 14 | + 14 | | + 14 | 41 |
| 7514/7521 |  | |  |  |  | -6 | | -6 | | |  |  | |  |  |  |  |  |  | |  |  |  |  |  |  |  |  | |  |  |  |  |  | |  | 2 |
| 7548/7549 |  | |  |  |  |  | |  | | |  |  | |  |  |  |  |  |  | |  |  |  |  |  |  |  | -2 | |  |  |  |  |  | | -2 | 2 |
| 7516 | **C** | |  |  |  |  | |  | | |  |  | |  |  |  |  |  |  | |  |  |  | A | G |  |  |  | |  |  |  |  |  | |  | 1/1 |
| 7538 | **T** | |  |  |  |  | |  | | |  |  | |  |  |  |  |  |  | |  |  |  |  |  |  |  |  | |  |  | G |  |  | |  | 1 |
| 7570 | **C** | |  |  |  |  | |  | | |  |  | |  |  |  |  | G |  | |  |  |  |  |  |  |  |  | |  |  |  |  |  | |  | 1 |
| 7588 | **A** | |  |  |  |  | |  | | |  |  | |  |  |  |  |  |  | |  |  |  |  |  |  |  |  | |  |  | G |  |  | |  | 1 |
| 7590 | **A** | |  |  |  |  | |  | | |  |  | |  |  |  |  |  |  | |  |  |  |  | G |  |  |  | |  |  |  |  |  | |  | 1 |
| 7591 | **C** | |  |  |  | T | |  | | |  |  | |  |  |  |  |  |  | |  |  |  |  |  |  |  |  | |  |  |  |  |  | |  | 1 |
| 7615 | **T** | |  |  |  |  | |  | | |  |  | |  |  |  |  |  |  | |  |  |  |  |  |  |  |  | |  |  | A |  |  | |  | 1 |
| 7617 | **A** | |  |  |  |  | |  | | | C | C | | C | C | C | C | C | C | | C | C | C | C | C | G | G | G | | G | G | G | G | G | | G | 31/10 |
| 7618 | **A** | |  |  |  | C | | C | | | C | C | | C | C | C | C | C | C | | C | C | C | C | C | C | C | C | | C | C | C | C | C | | C | 43 |
| 7623 | **C** | |  |  |  | A | | A | | | A | A | | A | A | A | A | A | A | | A | A | A | A | A | A | A | A | | A | A | A | A | A | | A | 43 |
| 7628 | **C** | |  |  |  | T | | T | | | T | T | | T | T | T | T | T | T | | T | T | T | T | T | T | T | T | | T | T | T | T | T | | T | 43 |
| 7631/7632 |  | |  |  |  |  | |  | | |  |  | |  |  |  |  |  |  | |  |  |  |  |  |  |  |  | |  |  | +1 |  |  | |  | 1 |
| 7655 | **G** | |  |  |  |  | |  | | |  |  | |  |  |  |  |  |  | |  |  |  |  |  |  |  |  | |  |  |  | A |  | |  | 1 |
| 7661 | **A** | |  |  |  | G | | G | | | G | G | | G | G | G | G | G | G | | G | G | G | G | G | G | G | G | | G | G | G | G | G | | G | 43 |
| 7673 | **C** | |  |  |  |  | |  | | |  |  | |  |  |  |  |  |  | |  |  |  |  |  |  | G | G | | G | G | G | G | G | | G | 9 |
| 7674 | **C** | |  |  |  |  | |  | | |  |  | |  |  |  | T |  |  | |  |  |  |  |  |  |  |  | |  |  |  |  |  | |  | 1 |
| 7679 | **A** | |  |  | C |  | |  | | |  |  | |  |  |  |  |  |  | |  |  |  |  |  |  |  |  | |  |  |  |  |  | |  | 2 |
| 7696 | **T** | |  |  |  | G | | G | | | G | G | | G | G | G | G | G | G | | G | G | G | G | G | G | G | G | | G | G | G | G | G | | G | 43 |
| 7700 | **C** | |  |  |  | G | | G | | | G | G | | G | G | G | G | G | G | | G | G | G | G | G | G | G | G | | G | G | G | G | G | | G | 43 |
| 7747 | **G** | |  |  | C | C | | C | | |  |  | |  |  |  |  |  |  | |  |  |  |  |  |  |  |  | | C | C | C |  |  | |  | 7 |
| 7748 | **C** | |  | A | A | A | | A | | | A | A | | A | A | A | A | A | A | | A | A | A | A | A | A | A | A | | A | A | A | A | A | | A | 47 |
| 7668 | **A** | |  |  |  |  | |  | | |  |  | |  |  |  |  |  |  | |  |  |  |  |  |  |  |  | |  |  | -1 |  |  | |  | 1 |
| 7812/7813 |  | |  |  |  | + 20 | | + 20 | | | + 20 | + 20 | | + 20 | + 20 | + 20 | + 20 | + 20 | + 20 | | + 20 | + 20 | + 20 | + 20 | + 20 | + 20 | + 20 | + 20 | | + 20 | + 20 | + 20 | + 20 | + 20 | | + 20 | 43 |
| 7840 | **A** | |  |  |  | C | | C | | |  |  | |  |  |  |  |  |  | |  |  |  |  |  |  |  |  | |  |  |  |  |  | |  | 2 |
| 7884 | **C** | |  |  |  | A | | A | | |  |  | |  |  |  |  |  |  | |  |  |  |  | A | A | A | A | | A | A | A | A | A | | A | 13 |
| 7909 | **G** | |  |  | C |  | |  | | |  |  | |  |  |  |  |  |  | |  |  |  |  |  |  |  |  | |  |  |  |  |  | |  | 2 |
| 7911 | **A** | |  |  |  |  | |  | | |  |  | |  |  |  |  |  |  | |  |  |  |  | G |  |  |  | |  |  |  |  |  | |  | 1 |
| 7925 | **A** | |  |  |  |  | |  | | |  |  | |  | C |  |  |  |  | |  |  |  |  |  |  |  |  | |  |  |  |  |  | |  | 1 |
| 7941/7942 |  | |  |  |  |  | |  | | |  |  | |  |  |  |  |  |  | |  |  | +3 |  |  |  |  |  | |  |  |  |  |  | |  | 2 |
| 7943 | **C** | |  |  |  |  | |  | | |  |  | |  |  |  |  |  |  | |  |  | T |  |  |  |  |  | |  |  |  |  |  | |  | 2 |
| 7945 | **C** | |  |  |  |  | |  | | |  |  | |  |  |  |  |  |  | |  |  | T |  |  |  |  |  | |  |  |  |  |  | |  | 2 |
| 7948 | **A** | |  |  |  |  | |  | | |  |  | |  |  |  |  |  |  | |  |  | T |  |  |  |  |  | |  |  |  |  |  | |  | 2 |
| 7950 | **C** | |  |  |  |  | |  | | |  |  | |  |  |  |  |  |  | |  |  | T |  |  |  |  |  | |  |  |  |  |  | |  | 2 |
| 7954 | **G** | |  | A | A | A | | A | | | A | A | | A | A | A | A | A | A | | A | A | A | A | A | A | A | A | | A | A | A | A | A | | A | 47 |
| 7983 | **A** | |  |  |  |  | |  | | |  |  | |  |  | G |  |  |  | |  |  |  |  |  |  |  |  | |  |  |  |  |  | |  | 1 |
| 2 | **T** | |  |  |  |  | |  | | |  |  | | G | G |  |  |  |  | |  |  |  |  |  |  |  |  | |  |  |  |  |  | |  | 2 |
| 13 | **C** | |  |  |  |  | | T | | |  |  | |  |  |  |  |  |  | |  |  |  |  |  |  |  |  | |  |  |  |  |  | |  | 1 |
| 14 | **C** | |  |  |  |  | | T | | |  |  | |  |  |  |  |  |  | |  |  |  |  |  |  |  |  | |  |  |  |  |  | |  | 1 |
| 17/18 |  | |  | +1 |  | +1 | | +1 | | | +1 | +1 | | +1 | +1 | +1 | +1 | +1 | +1 | | +1 | +1 | +1 | +1 | +1 | +1 | +1 | +1 | | +1 | +1 | +1 | +1 | +1 | | +1 | 45 |
| 19/23 |  | |  | -3 |  |  | |  | | |  |  | |  |  |  |  |  |  | |  |  |  |  |  |  |  |  | |  |  |  |  |  | |  | 2 |
| 22 | **A** | |  |  |  |  | |  | | |  |  | |  |  |  |  |  |  | |  |  |  |  |  |  |  | C | |  |  |  |  |  | |  | 1 |
| **Lesion Type** | | | | | | | | | | | | | | | | | | | | | | | | | | | | | | | | | | | | | |
| Anal cancer | |  | |  | 1 | |  | |  | 1 | | 1 |  | |  |  |  |  | |  |  |  |  |  |  |  |  | | 1 |  |  |  |  |  |  | |  |
| Cervical Cells | |  | |  |  | |  | |  | 2 | | 1 | 1 | |  |  |  |  | |  |  |  |  |  |  |  |  | |  |  |  |  |  |  |  | |  |
| Genital Warts | | 1 | |  |  | | 1 | | 1 | 12 | | 1 |  | | 1 | 1 | 1 | 1 | | 1 | 1 | 1 | 2 | 1 | 1 |  | 1 | |  | 1 | 1 |  |  | 2 | 1 | |  |
| RRP | |  | | 2 | 1 | |  | |  | 1 | |  |  | |  |  |  |  | |  |  |  |  |  |  | 1 | 1 | |  |  |  | 1 | 1 |  |  | |  |
| **Total** | | **1** | | **2** | **2** | | **1** | | **1** | **16** | | **3** | **1** | | **1** | **1** | **1** | **1** | | **1** | **1** | **1** | **2** | **1** | **1** | **1** | **2** | | **1** | **1** | **1** | **1** | **1** | **1** | **1** | |  |
| **P Values for association with anogenital lestion** | |  | |  |  | |  | |  | **0.23** | |  |  | |  |  |  |  | |  |  |  |  |  |  |  |  | |  |  |  |  |  |  |  | |  |

Nucleotide positions given are from the reference sequence HPV 6b (GenBank Acc. No X00203). Variant groups are assigned by HPV6 lineage A, and lineage B which is denoted according to sublineages B3 and B1. The frequency (Freq) indicates the number of isolates for each variant identified across all HPV6 variant groups. * A 19bp deletion was observed at nucleotide position 7365/7385 in the sublineage B1 reference genome only, with this being the only variation between the sublineage B1 reference and B1-1. + denotes an insertion. – denotes a deletion. RRP refers to recurrent respiratory papillomatosis. Two-tailed P values were calculated using Fisher exact test.
