# Supplementary material for: Human Papillomavirus Type 6 and 11 Genetic Variants Found in 71 Oral and Anogenital Epithelial Samples from Australia
Source: PLoS One. 2013 May 17;8(5):e63892. doi: 10.1371/journal.pone.0063892 (PMC3656832; doi:10.1371/journal.pone.0063892)
Supplement: Table S6 — HPV11 nucleotide and amino acid sequence variation in the ORF of E6 and E7 from 22 clinical isolates representing four different lesion types. (DOCX) [file pone.0063892.s006.docx]

**Table S6.** HPV11 nucleotide and amino acid sequence variation in the ORF of E6 and E7 from 22 clinical isolates representing four different lesion types.

|  |  |  | **HPV11 E6/E7 Variant Groups** | | |  |  |
| --- | --- | --- | --- | --- | --- | --- | --- |
| **Nucleotide Position** | **ORF** | **Ref**  **M14119** | **A2-1** | **A2-2** | **A2-3** | **Amino Acid** | **Frequency** |
| 137 | **E6** | **T** | C | C | C |  | 22 |
| 380 | **E6** | **C** | T | T |  |  | 21 |
| 383 | **E6** | **T** |  |  | G | N94K | 1 |
| 392 | **E6** | **T** |  |  | A |  | 1 |
| 398 | **E6** | **A** |  |  | C | K99N | 1 |
| 466 | **E6** | **G** |  | A |  | G122E | 1 |
| 662 | **E7** | **G** | T | T |  | A45S | 21 |
| 761 | **E7** | **C** |  |  | A | Q78K | 21 |
| **Lesion Type** |  |  |  |  |  |  |  |
| Anal cancer | | | 2 |  |  |  |  |
| Cervical Cells | | | 1 |  |  |  |  |
| Genital Warts | | | 8 | 1 | 1 |  |  |
| Recurrent Respiratory Papillomatosis | | | 9 |  |  |  |  |
| **Total** | | | **20** | **1** | **1** |  |  |
| **P Values for association with anogenital lestion** | | | **0.49** |  |  |  |  |

Nucleotide positions given are from the HPV11 sublineage A1 reference sequence (GenBank Acc. No M14119). Variant groups are denoted according to sublineage A2. The frequency indicates the number of isolates for each variant identified across all HPV11 variant groups. Two-tailed P values were calculated using Fisher exact test.
