# Supplementary material for: Human Papillomavirus Type 6 and 11 Genetic Variants Found in 71 Oral and Anogenital Epithelial Samples from Australia
Source: PLoS One. 2013 May 17;8(5):e63892. doi: 10.1371/journal.pone.0063892 (PMC3656832; doi:10.1371/journal.pone.0063892)
Supplement: Table S7 — HPV11 nucleotide sequence variation in the LCR from 22 clinical isolates representing four different lesion types. (DOCX) [file pone.0063892.s007.docx]

**Table S7**. HPV11 nucleotide sequence variation in the LCR from 22 clinical isolates representing four different lesion types.

|  |  | **HPV11 LCR Variant Groups** | | | | | | | | | | |  |
| --- | --- | --- | --- | --- | --- | --- | --- | --- | --- | --- | --- | --- | --- |
| **Nucleotide Position** | **Ref M14119** | **A2-1** | **A2-2** | **A2-3** | **A2-4** | **A2-5** | **A2-6** | **A2-7** | **A2-8** | **A2-9** | **A2-10** | **A2-11** | **Freq** |
| 7302 | **A** | C |  |  |  |  |  |  |  |  |  |  | 1 |
| 7333 | **C** | G |  |  |  |  |  |  |  |  |  |  | 1 |
| 7359 | **T** |  |  |  |  |  |  |  |  |  |  | G | 1 |
| 7387/7391 |  |  |  |  |  |  |  |  |  |  | -3 |  | 1 |
| 7413 | **A** |  |  |  |  |  | C | C | C | C | C | C | 17 |
| 7479 | **C** |  | T | T | T | T | T | T | T | T | T | T | 21 |
| 7505/7507 |  |  | -1 | -1 | -1 | -1 | -1 | -1 | -1 | -1 | -1 | -1 | 21 |
| 7547 | **T** | C | C | C | C | C | C | C | C | C | C | C | 22 |
| 7575/7576 |  |  |  | +1 |  |  | +2 | +1 | +1 |  | +1 | +1 | 16/1 |
| 7587 | **T** |  |  |  | G | G |  |  |  |  |  |  | 2 |
| 7625 | **C** |  |  |  | T |  |  |  |  |  |  |  | 1 |
| 7626 | **A** | C |  |  |  |  |  |  |  |  |  |  | 1 |
| 7682 | **T** | G |  |  |  |  |  |  |  |  |  |  | 1 |
| 7819 | **A** |  |  |  | C | C | -1 | -1 |  |  |  |  | 2/2 |
| 7902 | **T** | C |  |  |  |  |  |  |  |  |  |  | 1 |
| 7926 | **C** | T |  |  |  |  |  |  |  |  |  |  | 1 |
| **Lesion Type** | |  |  |  |  |  |  |  |  |  |  |  |  |
| Anal cancer | |  |  |  |  | 1 |  |  | 1 |  |  |  |  |
| Cervical Cells | |  |  |  |  |  |  |  |  |  |  | 1 |  |
| Genital Warts | | 1 |  | 1 |  |  |  |  | 7 |  | 1 |  |  |
| RRP | |  | 1 |  | 1 |  | 1 | 1 | 4 | 1 |  |  |  |
| **Total** | | **1** | **1** | **1** | **1** | **1** | **1** | **1** | **12** | **1** | **1** | **1** |  |
| **P Values for association with anogenital lestion** | |  |  |  |  |  |  |  | **0.66** |  |  |  |  |

Nucleotide positions given are from the HPV11 sublineage A1 reference sequence (GenBank Acc. No M14119). Variant groups are denoted according to sublineage A2. The frequency indicates the number of isolates identified across all HPV11 genomic variant groups. RRP refers to respiratory papillomatosis. + denotes an insertion. – denotes a deletion. Two-tailed P values were calculated using Fisher exact test.
