# Supplementary material for: Correction: Human Papillomavirus Type 6 and 11 Genetic Variants Found in 71 Oral and Anogenital Epithelial Samples from Australia
Source: PLoS One. 2015 Feb 6;10(2):e0117962. doi: 10.1371/journal.pone.0117962 (PMC4319825; doi:10.1371/journal.pone.0117962)
Supplement: S2 File — (PDF) [file pone.0117962.s002.pdf]

PLOS ONE

Human Papillomavirus Type 6 and 11 Genetic Variants Found in 71 Oral and Anogenital Epithelial Samples from Australia

Jennifer A. Danielewski[1,2], Suzanne M. Garland[1,2,3], Jenny McCloskey[4,5], Richard J. Hillman[6], Sepehr N. Tabrizi[1,2,3]*

1 Department of Microbiology and Infectious Diseases, Royal Women's Hospital, Melbourne, Australia, **2** Murdoch Childrens Research Institute, Melbourne, Australia, **3** Department of Obstetrics and Gynaecology, University of Melbourne, Melbourne, Australia, **4** Sexual Health Services, Royal Perth Hospital, Perth, Australia, **5** School of Pathology and Laboratory Medicine, University of Western Australia, Perth, Australia, **6** Western Sydney Sexual Health Centre, University of Sydney, Sydney, Australia

Abstract

Genetic variation of 49 human papillomavirus (HPV) 6 and 22 HPV11 isolates from recurrent respiratory papillomatosis (RRP) (n = 17), genital warts (n = 43), anal cancer (n = 6) and cervical neoplasia cells (n = 5), was determined by sequencing the long control region (LCR) and the E6 and E7 genes. Comparative analysis of genetic variability was examined to determine whether different disease states resulting from HPV6 or HPV11 infection cluster into distinct variant groups. Sequence variation analysis of HPV6 revealed that isolates cluster into variants within previously described HPV6 lineages, with the majority (65%) clustering to HPV6 sublineage B1 across the three genomic regions examined. Overall 72 HPV6 and 25 HPV11 single nucleotide variations, insertions and deletions were observed within samples examined. In addition, missense alterations were observed in the E6/E7 genes for 6 HPV6 and 5 HPV11 variants. No nucleotide variations were identified in any isolates at the four E2 binding sites for HPV6 or HPV11, nor were any isolates found to be identical to the HPV6 lineage A or HPV11 sublineage A1 reference genomes. Overall, a high degree of sequence conservation was observed between isolates across each of the regions investigated for both HPV6 and HPV11. Genetic variants identified a slight association with HPV6 and anogenital lesions (p = 0.04). This study provides important information on the genetic diversity of circulating HPV 6 and HPV11 variants within the Australian population and supports the observation that the majority of HPV6 isolates cluster to the HPV6 sublineage B1 with anogenital lesions demonstrating an association with this sublineage (p = 0.02). Comparative analysis of Australian isolates for both HPV6 and HPV11 to those from other geographical regions based on the LCR revealed a high degree of sequence similarity throughout the world, confirming previous observations that there are no geographically specific variants for these HPV types.

Citation: Danielewski JA, Garland SM, McCloskey J, Hillman RJ, Tabrizi SN (2013) Human Papillomavirus Type 6 and 11 Genetic Variants Found in 71 Oral and Anogenital Epithelial Samples from Australia. PLoS ONE 8(5): e63892. doi:10.1371/journal.pone.0063892

Editor: Robert D. Burk, Albert Einstein College of Medicine, United States of America

Received December 26, 2012; **Accepted** April 7, 2013; **Published** May 17, 2013

Copyright: © 2013 Danielewski et al. This is an open-access article distributed under the terms of the Creative Commons Attribution License, which permits unrestricted use, distribution, and reproduction in any medium, provided the original author and source are credited.

Funding: This study was supported through Royal Women's Hospital Departmental funding. No additional funding was received for this study. The funders had no role in study design, data collection and analysis, decision to publish, or preparation of the manuscript.

Competing Interests: The authors have declared that no competing interests exist.

* Sepehr.Tabrizi@thewomens.org.au

Introduction

Human papillomaviruses (HPVs) have been demonstrated to cause a diverse range of epithelial lesions. To date, 120 HPV's have been identified and each of these types are associated with infection at particular anatomical sites [1,2]. HPV6 and HPV11 are the most prevalent 'low-risk' alpha-papillomavirus types and are commonly associated with genital warts. Several recent studies detected HPV6 and/or HPV11 in 92.3% (n = 351 females) [3] and 92.4% (n = 422 males and females) [4] in biopsy confirmed anogenital warts, with HPV6 being significantly more often associated with anogenital warts than HPV11 [3,4]. Interestingly, gender bias has been associated with HPV11 genital warts whereby the proportion of HPV11 genital warts is three times higher in males than females [4]. Reported median intervals from infection of HPV6 and/or HPV11 to clinical detection of genital warts are 6 and 4.9 months respectively [3], although infection to development of lesions can range from 2.8 up to 8 months for these HPV types [5,6]. Worldwide, genital warts are one of the most common sexually transmitted diseases [7,8] and although not life threatening they cause significant morbidity and personal distress to those affected [9]. In addition, they are a risk factor for development of intraepithelial neoplasia [10].

Although the majority of HPV related non-malignant diseases are attributable to HPV 6 and 11 [11], these types have also been associated with epithelial lesions which can have a more aggressive pathology, such as the rare benign hyperproliferative lesions of recurrent respiratory papillomatosis (RRP) which causes significant morbidity and mortality, as well as a small number of Buschke-Löwenstein tumours [12], anal [13], vulvar [14] and cervical cancers [15]. RRP generally presents with progressive hoarseness and stridor related to growth of multiple exophytic lesions within the larynx, though lesions can also occur at other sites within the oropharyngeal and lower respiratory tract [16]. RRP occurs in both juveniles and adults, with the route for HPV exposure to newborns thought to be predominantly via maternal genital infection, although the frequency and routes for both

vertical and horizontal transmission remain controversial [17]. The pathological course of this disease can vary from spontaneous remission, relatively stable lesions, to aggressive cases which can be potentially life threatening due to the number and size of lesions causing severe respiratory obstruction [17,18]. For this reason, juvenile RRP can have a devastating impact on affected children due to the number and frequency of surgical procedures required to control their disease, with some patients requiring hundreds of operations in their lifetime. Malignant transformation to squamous cell carcinoma in RRP is a rare outcome and is often a consequence of irradiation, bleomycin chemotherapy or cigarette smoking, although it can also develop without any known carcinogenic risk factors [19].

Anal cancer is uncommon in the general population, although incidence in the general Australian population is increasing [20]. In women the incidence of anal cancer is even higher than in men, although the rate of annual increase is almost two-fold higher in Australian men (3.42% in men: 1.88% in women) [20], with rates also increasing in European and US men [11,21]. The incidence of anal cancer is greater in high risk populations such as men who have sex with men (MSM), women with previous HPV-related genital or cervical neoplasias and immunosuppressed individuals such as those with HIV infection [20,21,22,23,24]. Additional risk factors include receptive anal intercourse, number of lifetime sexual partners, smoking, genital warts, immunosuppression and HPV infection [22,25], of which HPV type 16 is the most prevalent in anal cancer [22,23,26]. Information regarding the prevalence of HPV types 6 and 11 in the anal canal, lesions of anal intraepithelial neoplasia (AIN) and anal cancer is limited [13,26,27]. The largest of these studies reported gender specific prevalence for invasive anal cancer (HPV6 at 3.4% in females and 0% in males; HPV11 at 3.4% in females and 8.3% in males), anal HSIL (HPV6 at 7% in females and 4.4% in males; HPV11 at 0% in females and 3.3% in males) and for anal LSIL (HPV6 at 22% in males only; HPV11 at 43% in males only) [26].

Cervical cancer and its precursors namely high grade cervical intraepithelial neoplasia (CIN2/3) and adenocarcinoma insitu (AIS) are frequently associated with persistent HPV infection of types HPV16 and HPV18 [28]. Although HPV6 and HPV11 are most commonly reported with normal cytology at a prevalence ranging from 1–1.8% for HPV6 and 0.6–20% for HPV11 [29,30,31], they have also been reported in association with precancerous cervical lesions and cervical cancer. A systematic review on the prevalence of HPV and cervical cancer reported a pooled prevalence of 4.5% for both HPV6 and HPV11 for CIN1, pooled prevalence of 0% for both HPV6 and HPV11 in cases of CIN2/3, and in cases of cervical carcinoma a pooled prevalence of 4.3% was reported for HPV6 and 0.1% for HPV11 [14]. A recent large global study reported the most prevalent HPV types associated with invasive cervical cancer as HPV16 (57%) and HPV18 16% throughout the world, whereas the overall prevalence of associated low risk types HPV6 (0.4%) and HPV11 (0.5%) are comparatively rare [15].

Several key gene regions and their role in malignant transformation have been extensively studied in high-risk HPV types 16 and 18. For example, viral E6 and E7 oncogenes are key mediators in cell transformation through the disruption of tumour suppressor Rb/E2F and subsequent p53 pathways respectively, which are critical in regulating the initiation of DNA replication. Loss of function or disruption of these pathways due to integration of HPV DNA, leading to increased expression of E6 and E7 proteins, has been demonstrated to result in the development of cervical carcinoma [32,33,34]. E6 and E7 expression levels have been demonstrated to play a key role in the progression of cervical carcinoma via deregulation of cellular genes controlling tumour cell proliferation [33]. Unlike high-risk HPV types which often integrate into the host genome but may also exists episomally, low-risk HPV types such as HPV6 and HPV11 are not known to integrate into the host DNA. Despite this, the role of E6 and E7 in HPV6 and HPV11 associated epithelial lesions has warranted investigation. A limited number of studies to date have investigated the association of genetic variants within these gene regions in lesions such as genital warts and RRP [35,36,37,38,39,40,41,42,43], with the majority of these studies being carried out on one lesion type or having small sample sizes. The long control region (LCR) is another key region which has been investigated in HPV6 and HPV11 associated epithelial lesions [35,36,37,40,41,42,43,44,45]. The LCR contains numerous highly conserved short motifs which act as key regulatory elements that govern gene expression and replication [46]. Among these are four E2 binding sites, which, in conjunction with an SP1 transcription factor binding site and a TATA box, are thought to modulate E6 and E7 promoter and transcription activity [46].

Previous studies investigating genetic variation of these gene regions have used standard reference genomes for comparative purposes. These standard reference genomes for HPV6 are denoted prototypic HPV6b, and non-prototypic HPV6a and HPV6vc, and one standard reference genome for HPV11. A recent study based on phylogenetic analysis of complete genomes derived from published HPV6 and HPV11 variants has proposed a new standard nomenclature for HPV6 and HPV11 [47]. Two deeply separate clades were observed for HPV6 with the reference genome HPV6b forming lineage A, and reference genomes HPV6a and HPV6vc forming lineage B. Within HPV6 lineage B, there are three sublineages with HPV6vc belonging to sublineage B1 and HPV6a belonging to sublineage B3. HPV11 variants were found to be more highly conserved, not meeting the criteria for classification into more than one lineage. The nomenclature proposed for the HPV11 lineage is based on two clades, referred to as sublineage A1 which includes variants clustering with the HPV11 reference genome, and sublineage A2 which includes all other variants. This new standard nomenclature has been adopted to refer to reference genomes used for this study.

This study examines the genetic variability of the LCR, E6 and E7 gene regions in both HPV6 and HPV11 across different lesion types to determine whether different disease states resulting from single HPV6 or HPV11 infection cluster into distinct variant groups. In addition, current data on the genetic diversity of HPV6 and HPV11 variants present within the Australian population are very limited. Only one study has been conducted within the Australian population, sequencing only a portion of the LCR for 47 HPV6 or HPV11 RRP isolates [44]. Consequently, given the limited number of descriptive studies both in Australia and worldwide investigating genetic variation in HPV6 and HPV11, this study aimed to contribute to the expanding knowledge about genetic diversity of these genomes by investigating various HPV 6 and 11 lesions within the Australian population.

Methods

Ethics Statement

All patients provided written informed consent at time of treatment whereby ethics approval was received from: the human research ethics committee at St Vincent's Hospital, Sydney, Australia for anal cancer biopsies, the health ethics research committee at the Royal Perth Hospital, Perth, Australia for genital wart biopsies, and the human research ethics committee at the Royal Women's Hospital, Melbourne, Australia for the collection

of cervical cells. In addition, ethics approval was obtained from the ethics in human research committee of the Royal Children's Hospital, Melbourne, Australia for juvenile RRP biopsies whereby written informed consent was provided by either the parent or guardian at time of treatment.

Clinical Samples

Based on single HPV infection status a total of 71 HPV 6 and HPV11 isolates from four different disease states being: RRP, genital warts, anal cancer, and cervical neoplasia cells were included in this study. Formalin fixed, paraffin embedded (FFPE) biopsies collected at the Royal Children's Hospital, Melbourne between 1964 and 1994 from 17 juvenile RRP patients described previously [48] were used. A total of 54 fresh wart biopsies from 43 patients were collected at Royal Perth Hospital in Perth, Australia from December 2007 to July 2010. Overall, twenty-seven of these were anal, 19 peri-anal, two vulval, three from the anal canal and the location was not specified for three wart samples. Of the 11 patients with duplicate samples, 9 had a peri-anal plus an anal biopsy, while two had a peri-anal plus a vulval biopsy. As patient information was incomplete for 2 of the wart biopsies, based on 41 wart biopsies four were HIV positive patients (two male and two female), 10 were heterosexual females, 7 were heterosexual males, 8 were bisexual males and 18 were MSM. In addition, a total of 6 anal cancer FFPE biopsies collected from November 1998 to February 2007 at St Vincent's Hospital in Sydney, Australia were included in this study. All 6 anal cancers were from male MSM patients. Five cervical cell samples were derived from a previous study as described in [49], whereby cytology reported two samples as normal, while histology reported two samples as low-grade cervical intraepithelial neoplasia (CIN1) and one sample as CIN2.

DNA Extraction

For FFPE archival tissue, 5 – 10 µm sections were deparaffinised with 800 µl histolene, then mixed with 400 µl absolute ethanol. The tissue was centrifuged at 14,000 × g for 2 minutes. The resultant pellet was washed with 1 ml of absolute ethanol, centrifuged for 2 minutes and the supernatant was discarded. The pellet was air dried and subsequently incubated for 4 hrs at 55°C with 160 µl tissue lysis buffer (Roche Diagnostics, Mannheim, Germany) and proteinase K at a final concentration of 1 mg/ml. DNA was then isolated from digested tissue using the MagNA Pure LC DNA Isolation Kit I (Roche Diagnostics) on the automated MagNA Pure LC extraction system, according to the manufacture's protocol, with the addition of 33.3 µg/ml of poly(A) RNA carrier to the lysis buffer which has been demonstrated to improve extraction efficiency. Nucleic acid was eluted into a final volume of 100 µl.

Fresh 1–2 mm biopsies were homogenised using MagNA Lyser (Roche Diagnostic) with 250 µl of Tissue Lysis Buffer (Roche Diagnostics), by two repetitions of 30 seconds at a speed of 7000 rpm in the MagNA Lyser Instrument. Proteinase K was added to a final concentration of 1 mg/ml, and samples were incubated at 55°C until fully digested. DNA was then isolated from the digested tissue as described above. DNA was isolated from the PreservCyt samples of cervical cells using the MagNA Pure LC DNA Isolation Kit I on the automated MagNA Pure LC extraction system, according to a modified protocol [50].

Classification of HPV Types

HPV genotyping of all archival FFPE tissues were confirmed by using the INNO-LiPA HPV genotyping test (Innogenetics, Ghent, Belgium) which has demonstrated increased sensitivity and accuracy for archival tissue [51]. Given the potential for reduced amplification efficiency due to fragmentation of the DNA template from archival paraffin embedded tissue, the INNO-LiPA HPV genotyping test which amplifies a short 65 bp region of the HPV L1 gene and detects 28 anogenital HPV types, was used as described in [51]. Fresh genital wart biopsy samples were genotyped for HPV using PapType (Genera Biosystems, Melbourne, Australia) which amplifies a region of approximately 160 bp of the HPV L1 gene and allows the identification of 16 anogenital HPV genotypes, as described in [52]. Cervical cells were HPV genotyped as described in [49]. Ultimately, all single HPV6 and HPV11 positive samples were sequenced as outlined below, further confirming the HPV genotype present.

Amplification of HPV 6 and 11 LCR, E6 and E7

DNA positive for HPV6 or HPV11 samples were subject to type and gene specific PCRs. All primers used for genetic variation analysis of HPV6 LCR, E6 and E7 (Table S3), were designed using Primer3 [53] and were based on the reference genome from HPV6 lineage A (GenBank Acc. No. X00203), in addition to a modified version of the LCR sequence which was amended by the inclusion of a 94 bp segment after nucleotide position 7350, as reported by [45]. HPV6 primers were also aligned against reference genomes from HPV6 lineage B, sublineage B3 (GenBank Acc. No. L41216) and sublineage B1 (GenBank Acc. No AF092932). The HPV11 sublineage A1 reference genome (GenBank Acc. No M14119) was used in conjunction with Primer3 [53] to design primers for the LCR, E6 and E7 used for genetic variation analysis of HPV11 (Table S3). Primers were designed for a maximum amplicon length of approximately 250 base pairs, to accommodate potentially fragmented archival tissue samples.

All PCR reactions were performed in a 50 µl reaction volume containing 0.5 µM of each primer, 2 mM of each deoxynucleoside triphosphate, 2.5 mM $MgCl_2$, 1 U AmpliTaq® Gold DNA Polymerase and 1X reaction buffer (Applied Biosystems, Carlsbad, California, U.S.), and 2.5 to 5 µl of DNA template. Following a denaturation step of 94°C for 9 min, PCR steps at 94°C for 30 sec, 55°C for 20 sec and 72°C for 30 sec for 45 cycles, followed by an extension step at 72°C for 10 min, on a GeneAmp PCR System 9700 thermocycler (Applied Biosystems). Amplicons were run on a 2% agarose gel for visualization, using 1000 base pair HyperLadder IV (BioLine, London, U.K.) for sizing.

Sequencing and Analysis of Genetic and Amino Acid Variation

Amplicons were sent to the Australian Genome Research Facility for purification and Sanger DNA sequencing using a AB3730xl capillary sequencer (Applied Biosystems). To minimise PCR and sequencing errors, sequencing was performed on both forward and reverse stands with predicted sequencing error of 0.01% based on sequencing of control DNA. For regions which did not meet our quality control criteria (short sequence reads, weak sequence whereby signal to noise ratios were low and in cases of poor peak resolution) confirmation of HPV sequence was carried out by repeating both the forward and reverse strands in duplicate. Sequences were assembled using SeqMan version 8 (DNASTAR, Madison, Wisconsin, U.S.) and aligned against reference genomes using SeaView version 4 [54]. Nucleotide and amino acid variation and phylogenetic analyses were conducted using MEGA5 [55]. HPV type 6 and 11 variants were defined as being distinct if one or more nucleotide variation was detected in the region analysed, relative to the reference genome and other isolates. For HPV6, the lineage A reference genome (GenBank

Acc. No. X00203) was used for nucleotide position numbering with the corrected LCR sequence as described by [45] to determine LCR variants. For comparison of genomic variation representative sequences from HPV6 lineage A (GenBank Acc. No. X00203) and B (sublineage B1 GenBank Acc. No. AF092932 and sublineage B3 GenBank Acc. No. L41216) were used. The HPV11 sublineage A1 reference genome (GenBank Acc. No. M14119), was used for comparison of genomic variation and nucleotide position numbering, corrected for a 2 bp insertion in the LCR at nucleotide position 7717–7718 [43,45].

Nucleotide Sequence Accession Numbers

The HPV6 nucleotide sequences for the LCR, E6, E7 gene regions are deposited in the GenBank database under the accession numbers: LCR sequences (KC300093-KC300140), E6 sequences (KC300141-KC300188, KC333888), E7 sequences (KC300189-KC300237).

The HPV11 nucleotide sequences for the LCR, E6, E7 gene regions are deposited in the GenBank database under the accession numbers: LCR sequences (KC329850-KC329871), E6 sequences (KC329872-KC329893), E7 sequences (KC329894-KC329915).

Statistics

Data in categories were evaluated using two-tailed Fisher's exact test.

Results

Classification of HPV Types

This study used samples with either HPV6 or HPV11 infection. Of the entire sample set for each lesion type a total of 49 specimens were identified as HPV6 isolates, that is 8 from RRP lesions, 33 genital warts, 4 anal cancer biopsy specimens and 4 cervical cell samples. In addition, a total of 22 specimens were identified as HPV11 isolates, that is 9 RRP lesions, 10 genital warts, 2 anal cancer biopsy specimens and 1 cervical cell sample (Table S1). For the 11 patients who had two genital wart biopsies taken, the same HPV type and identical sequence was observed between the two isolates from the same patient within the E6/E7 ORFs. Therefore only one biopsy for each of these 11 patients is included in this study. Throughout the analysis only one HPV type and variant was found in each single lesion. Overall HPV 6 was slightly more associated with anogenital lesions ($p = 0.036$).

HPV6 Genomic Variants

All 49 HPV6 isolates were successfully amplified and sequenced across the E6 (nt 102–554) and E7 (nt 530–826) ORFs. For HPV6 E6/E7, a total of 18 single nucleotide variations (13 in the E6 and 5 in the E7 ORF) from the lineage A reference genome were identified (Table S2). No isolates were identical to the lineage A reference genome, however, a total of 5 isolates were identified as lineage A variants. Variants A-1 and A-2 were detected in 4 and 1 isolates respectively. All other isolates were identified as variants of HPV6 lineage B (Table S4). Twelve isolates grouped with sublineage B3, forming 4 variant groups, none of which were identical to the sublineage B3 reference sequence. Variant B3-1 had a single nucleotide variation from the sublineage B3 reference sequence, while variant B3–4 had three single nucleotide variations, and B3-2 and B3–3 each had 4 single nucleotide variations from the sublineage B3 reference sequence. Variants B3-2,-3 and -4 had the nucleotide A at position 791, as did the lineage A reference sequence, which is why no variation is marked in Table S4 at this position. Six of the 12 isolates were B3–4 variants. The majority of isolates, 26 in total, were identical to the sublineage B1 reference sequence. An additional 5 sublineage B1 variants were identified, each with a single nucleotide variation to the sublineage B1 reference sequence. There was a significant association with anogenital lesions with sublineage B1 ($p = 0.02$). Only one isolate was identified for variants B1–1 to B1–4, and two isolates for variant B1–5 (Table S4). Of the 18 single nucleotide variations there were 6 missense (non-synonymous) alterations, 3 in each of the E6 and E7 genes, and 12 silent (synonymous) alterations (Table S4). Four of these missense alterations (E19K and H50Q in E6, and F52Y and N88D in E7), have been reported previously [36,37,38,39,42].

A total of 48 HPV6 isolates were successfully amplified and sequenced across the LCR (nt 7292–7902, 1–101). Single nucleotide variations from the lineage A reference genome were identified at 45 sites across the LCR. In addition, 5 insertions and 4 deletions were identified (Table S2). Three variants within a total of 5 isolates grouped with the lineage A reference genome. No isolates were identified with identical sequence to the lineage A reference genome; however, LCR variant A-1 deviated by only one single nucleotide variation. Two variant groups of sublineage B3 were identified. Variant B3-1 contained a single nucleotide variation (one isolate), while variant B3-2 contained 2 single nucleotide variations (one isolate). The majority of isolates (41) grouped with the sublineage B1 forming a large number of variant groups, in total 21 (Table S5). The sublineage B1 reference sequence contains a 19 base pair deletion between nucleotide positions 7365 and 7385. This 19 base pair deletion was not observed in any of the sublineage B1 variants, with this being the only sequence variation from the sublineage B1 reference sequence for the variant group B1–1. All other sublineage B1 variants had one or more single nucleotide variations, insertions and deletions, with variant B1–19 having 10 single nucleotide variations, one insertion and one deletion. No nucleotide variations were observed at any of the four E2 binding sites for any of the HPV6 isolates. One RRP isolate was found to be the most variable across all HPV6 regions sequenced, with 9 single nucleotide variations and 2 amino acid changes E19K and H50Q for E6, and 9 single nucleotide variations, 1 insertion and 1 deletion for the LCR. None of the HPV6 E6/E7 or LCR genomic variants were specific to lesion type.

A total of 105 HPV6 LCR sequences were used to perform a comparative phylogenetic analysis, which included 48 Australian isolates from this study, 45 Slovenian [31] and 12 South African [41] publicly available sequences. Comparative nucleotide sequence analysis for the LCR region revealed a total of 38 variant groups across the three geographical sites (Figure 1). Six of these variant groups (A-1, A-4, B3-2, B1–2, B1–8, B1–23) shared identical nucleotide sequence between geographical regions. Of these 38 variants, 4 clustered with the HPV6 lineage A, although none were identical to this reference genome. The majority of HPV6 variants clustered to lineage B, with 5 sublineage B3 variants and 29 sublineage B1 variants. The sublineage B1 reference genome contains a 19 base pair deletion which was also not observed in any of B1 variants from Slovenia or South Africa.

HPV11 Genomic Variants

All 22 HPV11 isolates were successfully amplified and sequenced across the E6 (nt 102–554) and E7 (nt 530–826) ORFs. Three genomic variants were identified across the E6/E7 ORFs for HPV11 (Table S6). None of these genomic variants corresponded to the HPV11 sublineage A1 reference sequence (GenBank Acc. No M14119), with a total of 8 single nucleotide variations (six in the E6 and two in the E7 ORF) identified (Table

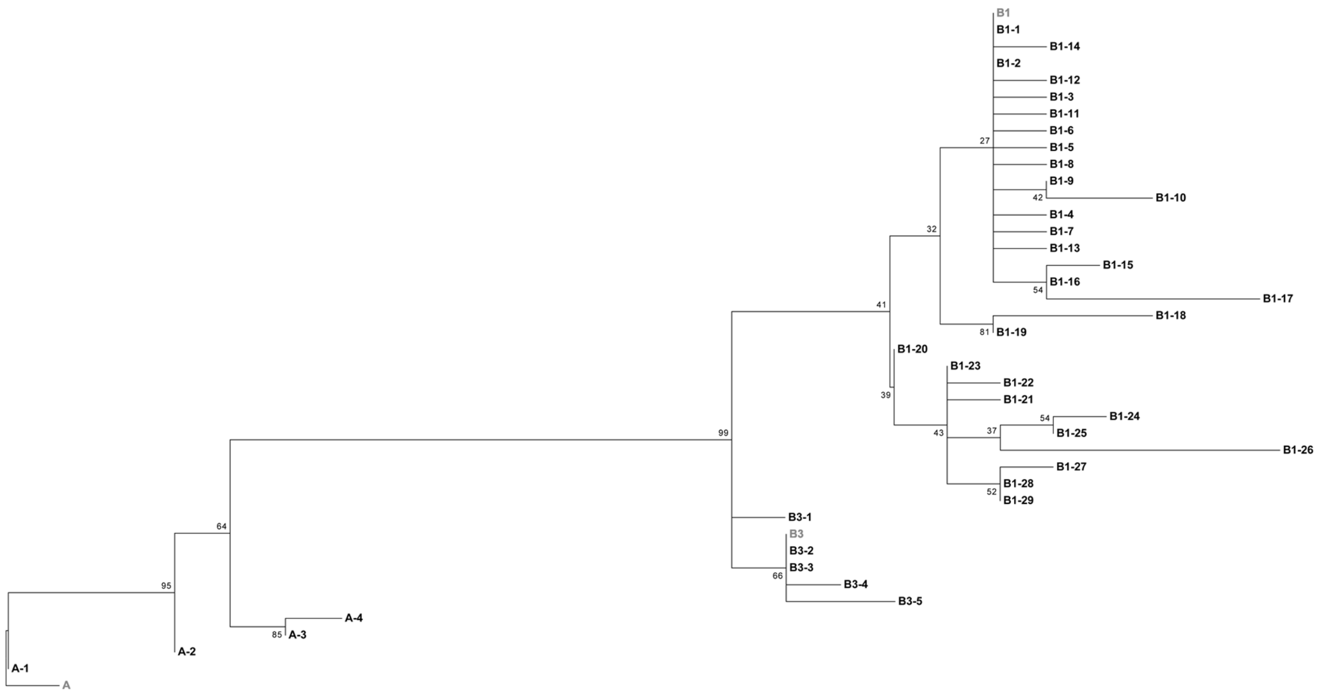

Figure 1. HPV6 LCR phylogenetic tree from Australia and other geographical regions. A maximum likelihood (ML) tree was inferred from molecular phylogenetic analysis of 105 aligned LCR sequences using MEGA5 based on the Tamura-Nei model with 500 bootstrap replicates [66]. These 105 sequences were derived from Australia (KC300093–KC300140, KC333888), Slovenia (FM876166–FM876210) and South Africa (JN573163–JN573174). Reference sequences are shown in grey for lineage A (X00203), and sublineages B1 (AF092932) and B3 (L41216). The 38 variants are named by the lineage and sublineage they cluster to. Lineage A was comprised of one Australian (A-2), one Slovenian (A-3) and two geographically shared (A-1, A-4) variants. Lineage B, specifically sublineage B1 was comprised of Australian (B1–4, B1–5, B1–9, B1–10, B1–12, B1–13, B1–14, B1–15, B1–16, B1–17, B1–18, B1–20, B1–21, B1–24, B1–25, B1–26, B1–27, B1–28, B1–29), Slovenian (B1–1, B1–3, B1–6, B1–7, B1–11, B1–19, B1–22) and geographically shared (B1–2, B1–8, B1–23) variants, and lineage B3 was comprised of Australian (B3–4, B3–5), South African (B3–1, B3–3) and geographically shared (B3–2). In cases where sequences were identical only one sequence from each counrty was use to represent each variant. In six instances variants from different geographical regions were identical; A–1, A–4 and B1–2 (Australia and Slovenia), B3–2 (Slovenia and South Africa), B1–8 and B1–23 (Australia, Slovenia and South Africa).
doi:10.1371/journal.pone.0063892.g001

S2). A total of 20 isolates grouped with variant A2–1. Variant A2–1 has three nucleotide alterations, which are identical to a genetic variant previously reported by [35] in a case of RRP (GenBank Acc. No FR872717). Variant A2–2 had a total of four nucleotide alterations, three of which were identical to that of A2–1; while variant A2–3 had a total of 5 nucleotide alterations, only one of which was identical to variant A2–1, each identified in one GW isolate only. Of the total 8 single nucleotide variations, 5 were identified as missense (non-synonymous) alterations, while the remaining 3 were silent (synonymous) alterations (Table S6). The missense alterations G122E in E6 and A45S in E7 have been reported previously [35,40,43].

The LCR from 22 HPV 11 isolates was successfully amplified and sequenced (nt 7277–7931, 1–101). Comparative analysis with the HPV11 sublineage A1 reference sequence revealed 13 single nucleotide variations, 1 insertion and 3 deletions in the LCR (Table S2). A total of 11 variants were identified for the LCR, each containing only one isolate with the exception of A2–8 which contained a total of 12 isolates (Table S7). No isolates had identical sequence to the sublineage A1 reference sequence with all variants having a minimum of 3 sequence variations. No nucleotide variations were observed in the HPV11 isolates at any of the four E2 binding sites. The isolate which was found to be the most variable across all HPV11 regions sequenced was a genital wart isolate (LCR A2–1) having 5 single nucleotide variations and 3 amino acid changes in the E6 (N94K, K99N) and E7 (Q78K) ORFs and 7 single nucleotide variations in the LCR. No HPV11 E6/E7 or LCR genomic variant was found to be specific to lesion type.

Comparative phylogenetic nucleotide sequence analysis was carried out on a total of 94 HPV11 LCR sequences. These publicly available sequences included 22 Australian isolates from this study, 63 Slovenian [43], 1 Chinese [56], 3 Hungarian [35] and 5 Thai [40] isolates. Comparative sequence analysis revealed a total of 21 variant groups (Figure 2). Only two of these variants clustered with the HPV11 sublineage A1 reference sequence. Variant A1–2 differed by one nucleotide variation from the HPV11 sublineage A1 reference sequence, while the A1–1 variant differed by two nucleotide variations. The remaining 19 variants clustered sublineage A2. Seven of these variants (A1–2, A2–4, A2–5, A2–8, A2–11, A2–18, A2–19) shared identical sequence from different geographical regions. Again the most variable isolate with 7 single nucleotide variations was the Australian variant A2–3 (Figure 2).

Discussion

We present one of the largest studies of Australian isolates to date which investigates genetic variation for HPV6 or HPV11 in some of the key HPV gene regions thought to be associated with development of lesions and disease progression in epithelial cells. This study compares genetic variation within various lesion types sampled from the Australian population for the two most prevalent

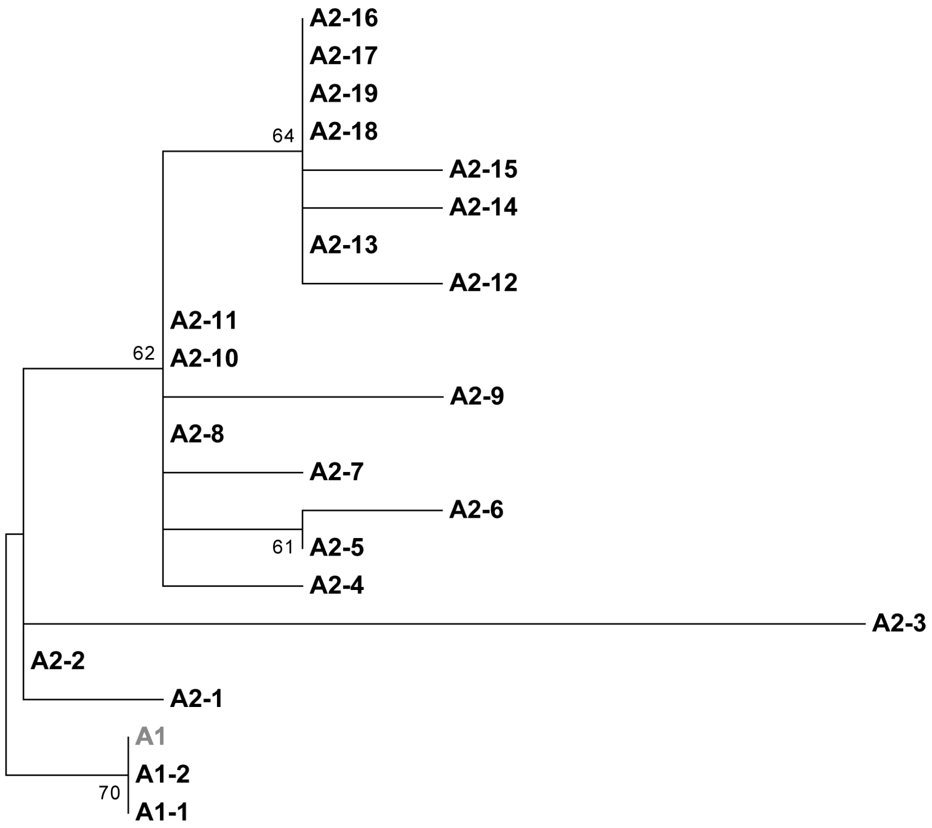

Figure 2. HPV11 LCR phylogenetic tree from Australia and other geographical regions. A maximum likelihood (ML) tree was inferred from molecular phylogenetic analysis of 94 aligned LCR sequences using MEGA5 based on the Tamura-Nei model with 500 bootstrap replicates [66]. These 94 sequences were derived from Australia (KC329850–KC329871), Slovenia (FN870625–FN870687), China (EU918768), Hungary (HE574701, HE574705, FR872717) and Thailand (JQ773408–JQ773412). The reference sequence (M14119) from sublineage A1 is shown in grey. The 21 variants are named by the lineage they cluster to. Sublineage A1 was comprised of one Slovenian variant (A1–1) and one geographically shared variant (A1–2). Sublineage A2 was comprised of 6 Australian (A2–3, A2–6, A2–13, A2–14, A2–16, A2–17), Slovenian(A2–1, A2–7, A2–10, A2–12, A2–15), Hungarian (A2–2), Thai(A2–9), and geographically shared (A2–4, A2–5, A2–8, A2–11, A2–18, A2–19) variants. In cases where sequences were identical only one sequence from that country was use to represent each variant. In seven instances variants from different geographical regions were also identical, again one sequence was used to represent each variant; A1–2 (China and Slovenia), A2–4 (Hungary and Thailand), A2–8 (Australia, Hungary and Slovenia), A2–5, A2–11, A2–18 (Australia and Slovenia), A2–19 (Australia, Slovenia and Thailand).
doi:10.1371/journal.pone.0063892.g002

types of low risk HPV associated with these diseases. Analysis of genetic variance revealed slight association with HPV6 and anogenital lesion and HPV11 and RRP with genetic variants HPV 6 B1 lineage being more strongly associated with anogenital lesion.

The majority of HPV11 isolates from this study clustered to the variant A2–1 for E6 and E7 (Table S6). This variant group encompassed all lesion types demonstrating no bias of particular variants to lesion type, and its sequence was identical for this region to that previously published [35]. Several descriptive studies recently reporting on genetic variation of E6 and E7 in populations derived from Hungary, Slovenia and Thailand [35,40,42,43] again demonstrate that HPV11 isolated from various lesions do not segregate based on lesion type. A previous Australian study which sequenced consecutive RRP lesions resulting from either HPV6 or HPV11 infection, found no significant association between HPV type, disease aggression or duration of RRP [44]. Gall et al. (2011), examined consecutive samples taken from the one case of HPV11 positive aggressive juvenile onset RRP at different time points and several different sites at each time point. Sequence analysis of the entire HPV11 genome revealed that each sample was identical, demonstrating conservation of the HPV11 genome throughout disease progression within that individual. A more recent study which followed 70 patients who had RRP for 1–22 years found identical sequence in follow up HPV6 and HPV11 samples, suggesting that RRP is the consequence of persistent infection with the initial HPV genomic variant [57]. Several studies have examined methylation associated with RRP whereby Gall et al. (2011) found all four E2 binding sites in the HPV11 LCR to be uniformly unmethylated, while the methylation status of all CpGs in the HPV6 LCR of aerodigestive tract papillomas were found to be unmethylated [58]. The results of this study, and others to date, suggest that specific disease pathology associated with these lesion types may result from more complex HPV and host interactions.

This study identified a series of variations in the E6 and E7 ORFs of the HPV6 genome within the Australian lesions sampled. A total of three amino acid alterations in each of the E6 and E7 ORFs of different HPV6 isolates were found, some of which were unique to a single isolate, while others were found in several or multiple cases. Of these, several unique amino acid alterations were identified: E113D in E6 and E73K in E7. Conversely, amino acid alterations E19K and H50Q in the E6 ORF and F52Y and N88D in the E7 ORF, present in numerous isolates, have been described previously [36,37,38,39,42], suggesting these are reasonably common in HPV6 throughout the world. Numerous

silent nucleotide alterations were also identified which have been previously reported [36,37,38,39,42]. For HPV11, this study identified 5 missense alterations, whereby all were unique, with the exception of the alteration at codon A45S in E7 [35,43]. The significance of these amino acid alterations is not currently known, and our data indicates that these are not specific to disease type. A previous study in which numerous amino acid changes were identified in the E7 and in particular the E6 ORFs, in addition to a duplication of enhancer elements in the LCR, did not reveal that any of these sequence features were unique to malignant versus benign tumours in numerous HPV6 lesion types [36]. Despite this, given the role that the E6 and E7 genes play in high risk HPV types 16 and18, and their clear role in the initiation of DNA replication in low risk HPV types, these amino acid alterations cannot be ruled out as playing a role in disease progression without further investigation.

When the prevalence of HPV-type in genital warts and RRP (lesions for which n>10) was considered, a type specific distribution was indicated for genital warts but not RRP. The prevalence of HPV 6 was found to be higher for genital warts (77% HPV6 vs 23% HPV11) in this sample set, as described previously by [4]. However, no difference in the prevalence of HPV6 (47%) and HPV11 (53%) was identified for Australian RRP samples, which may be reflective of the Australian population or due to sample size. Previous studies on prevalence of HPV type 6 or 11 associated with RRP have provided mixed reports. Some studies have reported no difference in prevalence between HPV6 or HPV11 in RRP [16], but found an association between HPV11 and more aggressive disease progression and/or cancer in RRP [16,59,60]. Whilst other studies, such as the recent large study by [4], found HPV6 (59%-HPV6 vs 28%-HPV11) was the most prevalent HPV type in RRP.

Variant analysis of the LCR within Australian isolates was completed for both HPV6 and HPV11. In recent years, a growing number of studies have described HPV6 LCR variations in isolates from the nucleotide positions of the lineage A reference genome [36,37,41,44,45]. Based on these nucleotide positions, the same single nucleotide alterations were observed at 6 [45] and 13 [37] shared locations with those of previous studies. The majority of HPV6 isolates grouped with the sublineage B1 reference sequence, forming a total of 22 variant groups. This reflects previous findings whereby the highest frequency of isolates and variants were identified for sublineage B1 [37,45]. Given the LCR contains a higher degree of variability than the combined E6/E7 gene region, the ability to distinguish between HPV6 sublineages B1 and B3 is limited for the E6/E7 gene region resulting in fewer Australian isolates and variant groups for HPV6 sublineage B3 in the LCR. Isolates sequenced in this study for the LCR in HPV11, revealed the same single nucleotide alterations at one [35,45] and, four [43,44] sites with previous studies. A similar number of HPV11-LCR variant groups were identified between this study (11) and (10) [45]. Despite the high number of variant groups identified for the LCR in both HPV6 and HPV11, the majority of these variant groups were made up of a single isolate. This may reflect the high degree of conservation observed in the LCR, and/or a low frequency of these variants circulating in the population. In addition, the four E2 transcription factor binding sites located within the LCR of HPV6 and HPV11 were found to be highly conserved, with no mutations identified in any of the isolates. Furthermore, a high degree of conservation was observed between isolates across each of the regions investigated for both HPV6 and HPV11. Normalising for the size of each region, the ratio of variable sites based on the most variable isolate for HPV6 E6, E7 and LCR were 0.7%, 0.7% and 1.3% respectively. While the ratio of variable sites based on the most variable isolate for HPV11 E6, E7 and LCR were 0.8%, 0.34% and 0.9% respectively. This study further demonstrates a high degree of conservation between isolates in these regions within the Australian population for these low risk HPV types. Further to this, Australian HPV6 variants identified within this study clearly demonstrated both HPV6 lineages A and B described by [47] with the majority of isolates clustering to sublineage B1. Australian HPV11 variants all clustered to sublineage A2.

Genomic regions such as the LCR have been used by previous studies to identify genetic variants as it provides enough genetic variation to differentiate between variants in both HPV6 and HPV11. For this reason, the LCR was selected for comparative phylogenetic sequence analysis between this and previous studies to determine whether genetic lineages previously described [35,37,40,41,42,43,47] are consistent between Australian and other geographical regions throughout the world. A comparative phylogenetic nucleotide analysis of Australian HPV6 variants to publicly available LCR sequences from Slovenia and South Africa further demonstrated a high level of conservation of this region throughout the world, with six instances of identical sequence observed between geographical regions, and the majority of variants clustering to HPV 6 lineage B, sublineage B1. Comparative phylogenetic sequence analysis of Australian isolates for the LCR of HPV11 with published LCR sequences from Solvenian, Chinese, Hungarian and Thai isolates demonstrated only several variants from Slovenia and China clustering to HPV11 sublineage A1, while the majority of variants clustered to sublineage A2. These results further support the high degree of sequence conservation between different geographical regions throughout the world, suggesting that geographically specific variants are not common for HPV6 or HPV11.

This current study also provides unique pre-vaccination data on the genetic variation present in RRP, anal cancer, cervical cells samples for HPV6 and HPV11 within the Australian population. For genital warts, based on age (14% female >26 years) and sex (74% male), at least 88% of these samples would have come from women and men ineligible for publically funded prophylactic HPV vaccination. The remaining 12% of genital warts were derived from women <26 years, whose vaccination status was unknown. The high proportion of genital wart isolates positive for HPV6 or HPV11 suggests that lesions developed either in unvaccinated patients, or in women who were vaccinated with prevalent infection. Australia was the first country to introduce a government-funded, population-based mass vaccination program in 2007 of the female population (12 to 26 years), using the quadrivalent vaccine Garadasil® (Merck, New Jersey, USA) targeting HPV types 16, 18, 6 and 11. It has already seen a reduction in the number of cases of genital warts due to a high coverage of vaccine [61,62]. For example, the Melbourne Sexual Health Centre reported that the proportion of women less than 28 years of age presenting with genital warts declined by 25% per quarter through 2008, irrespective of their vaccination status [63], and genital warts are now rarely seen in women under the age of 25 years [64]. A later study involving eight sexual health clinics Australia-wide found that there was no change in the proportion of women or heterosexual men diagnosed with genital warts before the vaccine program, whereas there was a substantial decline in diagnosed cases of genital warts in women 12–26 years of age [65]. Although the vaccine was licensed for boys 9–15 years of age, males were not eligible for the government program, so very few men/boys have received the vaccine. Despite this, the incidence of genital warts in young (<26 years) heterosexual men appears to be declining, thought to be due to the effect of herd immunity from

female vaccination. There has, however, been no reduction in the rates of anogenital warts in MSM or older heterosexual men [65]. The results of our study suggest that there is no causal association between specific variants and lesion type. This, in addition to the high level of sequence conservation observed for these low risk HPV types between variants throughout the world, implies the vaccine is unlikely to have an impact on the distribution of circulating types throughout the world.

In summary, we found slight association between HPV6 with anogenital lesions with sublineage B1 being predominate. This is consistent with the findings of other studies and suggests that viral and/or host epigenetic and other host factors (such as those associated with immune response) may play significant roles in disease susceptibility and progression. In addition, larger studies investigating whole genome sequences may shed further light on possible associations with pathogenesis. However, this study makes a significant contribution toward the slowly expanding knowledge about HPV6, and in particular HPV11 genomic diversity and pre-vaccination circulating HPV6/11 variants within the Australian population.

Supporting Information

Table S1 Summary of HPV6 and HPV11 lesion types.
(DOCX)

Table S2 Summary of genetic variation identified across all lesion types for HPV6 and HPV11.
(DOCX)

Table S3 Primers used for amplifying and sequencing the LCR, E6 and E7 ORFs.
(DOCX)

Table S4 HPV6 nucleotide and amino acid sequence variation in the ORF of E6 and E7 from 49 clinical isolates representing four different lesion types.
(DOCX)

Table S5 HPV6 nucleotide sequence variation in the LCR from 48 clinical isolates representing four different lesion types.
(DOCX)

Table S6 HPV11 nucleotide and amino acid sequence variation in the ORF of E6 and E7 from 22 clinical isolates representing four different lesion types.
(DOCX)

Table S7 HPV11 nucleotide sequence variation in the LCR from 22 clinical isolates representing four different lesion types.
(DOCX)

Acknowledgments

Dr Matt Stevens for the design of E6 and E7 primers used in this study. Angela Hurley for Paptype genotyping of the genital wart isolates. Professor Katja Seme for providing the modified HPV6b (GenBank Acc. No X00203) with a 94 bp insert used as the HPV6b reference sequence for this study. Dr Jimmy Twin for technical guidance and support with analysis.

Author Contributions

Conceived and designed the experiments: JAD SMG SNT. Performed the experiments: JAD. Analyzed the data: JAD. Contributed reagents/materials/analysis tools: SMG JM RJH SNT. Wrote the paper: JAD SMG JM RJH SNT.

References

1. Doorbar J, Griffin H (2007) Intrabody strategies for the treatment of human papillomavirus-associated disease. Expert Opin Biol Ther 7: 677–689.
2. Bernard HU, Burk RD, Chen Z, van Doorslaer K, Hausen H, et al. (2010) Classification of papillomaviruses (PVs) based on 189 PV types and proposal of taxonomic amendments. Virology 401: 70–79.
3. Garland SM, Steben M, Sings HL, James M, Lu S, et al. (2009) Natural history of genital warts: analysis of the placebo arm of 2 randomized phase III trials of a quadrivalent human papillomavirus (types 6, 11, 16, and 18) vaccine. J Infect Dis 199: 805–814.
4. Komlos KF, Kocjan BJ, Kosorok P, Luzar B, Meglic L, et al. (2012) Tumor-specific and gender-specific pre-vaccination distribution of human papillomavirus types 6 and 11 in anogenital warts and laryngeal papillomas: a study on 574 tissue specimens. J Med Virol 84: 1233–1241.
5. Oriel JD (1971) Natural history of genital warts. Br J Vener Dis 47: 1–13.
6. Winer RL, Kiviat NB, Hughes JP, Adam DE, Lee SK, et al. (2005) Development and duration of human papillomavirus lesions, after initial infection. J Infect Dis 191: 731–738.
7. Fairley CK, Donovan B (2010) What can surveillance of genital warts tell us? Sex Health 7: 325–327.
8. Scarbrough Lefebvre CD, Van Kriekinge G, Goncalves MA, de Sanjose S (2011) Appraisal of the burden of genital warts from a healthcare and individual patient perspective. Public Health 125: 464–475.
9. Pirotta M, Ung L, Stein A, Conway EL, Mast TC, et al. (2009) The psychosocial burden of human papillomavirus related disease and screening interventions. Sex Transm Infect 85: 508–513.
10. McCloskey JC, Metcalf C, French MA, Flexman JP, Burke V, et al. (2007) The frequency of high-grade intraepithelial neoplasia in anal/perianal warts is higher than previously recognized. Int J STD AIDS 18: 538–542.
11. Hartwig S, Syrjanen S, Dominiak-Felden G, Brotons M, Castellsague X (2012) Estimation of the epidemiological burden of human papillomavirus-related cancers and non-malignant diseases in men in Europe: a review. BMC Cancer 12: 30.
12. Lehn H, Ernst TM, Sauer G (1984) Transcription of episomal papillomavirus DNA in human condylomata acuminata and Buschke-Lowenstein tumours. J Gen Virol 65 (Pt 11): 2003–2010.
13. Palefsky JM, Giuliano AR, Goldstone S, Moreira ED, Jr., Aranda C, et al. (2011) HPV vaccine against anal HPV infection and anal intraepithelial neoplasia. N Engl J Med 365: 1576–1585.
14. Insinga RP, Liaw KL, Johnson LG, Madeleine MM (2008) A systematic review of the prevalence and attribution of human papillomavirus types among cervical, vaginal, and vulvar precancers and cancers in the United States. Cancer Epidemiol Biomarkers Prev 17: 1611–1622.
15. Li N, Franceschi S, Howell-Jones R, Snijders PJ, Clifford GM (2011) Human papillomavirus type distribution in 30,848 invasive cervical cancers worldwide: Variation by geographical region, histological type and year of publication. Int J Cancer 128: 927–935.
16. Wiatrak BJ, Wiatrak DW, Broker TR, Lewis L (2004) Recurrent respiratory papillomatosis: a longitudinal study comparing severity associated with human papilloma viral types 6 and 11 and other risk factors in a large pediatric population. Laryngoscope 114: 1–23.
17. Syrjanen S (2010) Current concepts on human papillomavirus infections in children. APMIS 118: 494–509.
18. Derkay CS, Wiatrak B (2008) Recurrent respiratory papillomatosis: a review. Laryngoscope 118: 1236–1247.
19. Rady PL, Schnadig VJ, Weiss RL, Hughes TK, Tyring SK (1998) Malignant transformation of recurrent respiratory papillomatosis associated with integrated human papillomavirus type 11 DNA and mutation of p53. Laryngoscope 108: 735–740.
20. Jin F, Stein AN, Conway EL, Regan DG, Law M, et al. (2011) Trends in anal cancer in Australia, 1982–2005. Vaccine 29: 2322–2327.
21. Johnson LG, Madeleine MM, Newcomer LM, Schwartz SM, Daling JR (2004) Anal cancer incidence and survival: the surveillance, epidemiology, and end results experience, 1973–2000. Cancer 101: 281–288.
22. Dona MG, Palamara G, Di Carlo A, Latini A, Vocaturo A, et al. (2012) Prevalence, genotype diversity and determinants of anal HPV infection in HIV-uninfected men having sex with men. J Clin Virol.
23. Machalek DA, Poynten M, Jin F, Fairley CK, Farnsworth A, et al. (2012) Anal human papillomavirus infection and associated neoplastic lesions in men who have sex with men: a systematic review and meta-analysis. Lancet Oncol.
24. Wentzensen N (2012) Screening for anal cancer: endpoints needed. Lancet Oncol.
25. Frisch M, Glimelius B, Wohlfahrt J, Adami HO, Melbye M (1999) Tobacco smoking as a risk factor in anal carcinoma: an antiestrogenic mechanism? J Natl Cancer Inst 91: 708–715.
26. Hoots BE, Palefsky JM, Pimenta JM, Smith JS (2009) Human papillomavirus type distribution in anal cancer and anal intraepithelial lesions. Int J Cancer 124: 2375–2383.

27. Komlos KF, Kocjan BJ, Kosorok P, Rus T, Toplak J, et al. (2011) Distribution of HPV genotypes in Slovenian patients with anal carcinoma: preliminary results. Acta Dermatovenerol Alp Panonica Adriat 20: 141–143.
28. Stevens MP, Tabrizi SN, Quinn MA, Garland SM (2006) Human papillomavirus genotype prevalence in cervical biopsies from women diagnosed with cervical intraepithelial neoplasia or cervical cancer in Melbourne, Australia. Int J Gynecol Cancer 16: 1017–1024.
29. Al-Awadhi R, Chehadeh W, Kapila K (2011) Prevalence of human papillomavirus among women with normal cervical cytology in Kuwait. J Med Virol 83: 453–460.
30. Panotopoulou E, Tserkezoglou A, Kouvousi M, Tsiaousi I, Chatzieleftheriou G, et al. (2007) Prevalence of human papillomavirus types 6, 11, 16, 18, 31, and 33 in a cohort of Greek women. J Med Virol 79: 1898–1905.
31. Stevens MP, Garland SM, Tan JH, Quinn MA, Petersen RW, et al. (2009) HPV genotype prevalence in women with abnormal pap smears in Melbourne, Australia. J Med Virol 81: 1283–1291.
32. Nevins JR (2001) The Rb/E2F pathway and cancer. Hum Mol Genet 10: 699–703.
33. Rosty C, Sheffer M, Tsafrir D, Stransky N, Tsafrir I, et al. (2005) Identification of a proliferation gene cluster associated with HPV E6/E7 expression level and viral DNA load in invasive cervical carcinoma. Oncogene 24: 7094–7104.
34. Scheffner M, Munger K, Byrne JC, Howley PM (1991) The state of the p53 and retinoblastoma genes in human cervical carcinoma cell lines. Proc Natl Acad Sci U S A 88: 5523–5527.
35. Gall T, Kis A, Feher E, Gergely L, Szarka K (2011) Virological failure of intralesional cidofovir therapy in recurrent respiratory papillomatosis is not associated with genetic or epigenetic changes of HPV11: complete genome comparison of sequential isolates. Antiviral Res 92: 356–358.
36. Grassmann K, Wilczynski SP, Cook N, Rapp B, Iftner T (1996) HPV6 variants from malignant tumors with sequence alterations in the regulatory region do not reveal differences in the activities of the oncogene promoters but do contain amino acid exchanges in the E6 and E7 proteins. Virology 223: 185–197.
37. Kocjan BJ, Poljak M, Cimerman M, Gale N, Potocnik M, et al. (2009) Prevaccination genomic diversity of human papillomavirus genotype 6 (HPV 6). Virology 391: 274–283.
38. Kovelman R, Bilter GK, Roman A, Brown DR, Barbosa MS (1999) Human papillomavirus type 6: classification of clinical isolates and functional analysis of E2 proteins. J Gen Virol 80 (Pt 9): 2445–2451.
39. Krige D, Mills HR, Berrie EL, Doherty NC, Jones DK, et al. (1997) Sequence variation in the early genes E1E4, E6 and E7 of human papilloma virus type 6. Virus Res 49: 187–191.
40. Chansaenroj J, Theamboonlers A, Junyangdikul P, Supiyaphan P, Poovorawan Y (2012) Whole genome analysis of human papillomavirus genotype 11 from cervix, larynx and lung. Asian Pac J Cancer Prev 13: 2619–2623.
41. Combrinck CE, Seedat RY, Randall C, Roodt Y, Burt FJ (2012) Novel HPV-6 variants of human papillomavirus causing recurrent respiratory papillomatosis in southern Africa. Epidemiol Infect 140: 1095–1101.
42. Kocjan BJ, Jelen MM, Maver PJ, Seme K, Poljak M (2011) Pre-vaccination genomic diversity of human papillomavirus genotype 6 (HPV 6): a comparative analysis of 21 full-length genome sequences. Infect Genet Evol 11: 1805–1810.
43. Maver PJ, Kocjan BJ, Seme K, Potocnik M, Gale N, et al. (2011) Prevaccination genomic diversity of human papillomavirus genotype 11: a study on 63 clinical isolates and 10 full-length genome sequences. J Med Virol 83: 461–470.
44. Gabbott M, Cossart YE, Kan A, Konopka M, Chan R, et al. (1997) Human papillomavirus and host variables as predictors of clinical course in patients with juvenile-onset recurrent respiratory papillomatosis. J Clin Microbiol 35: 3098–3103.
45. Heinzel PA, Chan SY, Ho L, O'Connor M, Balaram P, et al. (1995) Variation of human papillomavirus type 6 (HPV-6) and HPV-11 genomes sampled throughout the world. J Clin Microbiol 33: 1746–1754.
46. O'Connor M, Chan SY, Bernard HU (1995) Transcription factor binding sites in the long control regions of genital HPVs.. In G Meyers, H U Bernard, H Delius, C Baker, J Icenogle, A Halpern, and C Wheeler (ed), Human papillomaviruses, 1995 compendium, part III-A. ed: Los Alamos National Laboratory, Los Alamos, N.Mex. 21–40.
47. Burk RD, Chen Z, Harari A, Smith BC, Kocjan BJ, et al. (2011) Classification and nomenclature system for human Alphapapillomavirus variants: general features, nucleotide landmarks and assignment of HPV6 and HPV11 isolates to variant lineages. Acta Dermatovenerol Alp Panonica Adriat 20: 113–123.
48. Somers GR, Tabrizi SN, Borg AJ, Garland SM, Chow CW (1997) Juvenile laryngeal papillomatosis in a pediatric population: a clinicopathologic study. Pediatr Pathol Lab Med 17: 53–64.
49. Moore EE, Danielewski JA, Garland SM, Tan J, Quinn MA, et al. (2011) Clearance of human papillomavirus in women treated for cervical dysplasia. Obstet Gynecol 117: 101–108.
50. Stevens MP, Rudland E, Garland SM, Tabrizi SN (2006) Assessment of MagNA pure LC extraction system for detection of human papillomavirus (HPV) DNA in PreservCyt samples by the Roche AMPLICOR and LINEAR ARRAY HPV tests. J Clin Microbiol 44: 2428–2433.
51. Tan SE, Garland SM, Rumbold AR, Tabrizi SN (2010) Human papillomavirus genotyping using archival vulval dysplastic or neoplastic biopsy tissues: comparison between the INNO-LiPA and linear array assays. J Clin Microbiol 48: 1458–1460.
52. Tan SE, Garland SM, Rumbold AR, Tabrizi SN (2011) Comparison of the INNO-LiPA and PapType assays for detection of human papillomavirus in archival vulva dysplasia and/or neoplasia tissue biopsy specimens. J Clin Microbiol 49: 3980–3982.
53. Rozen S, Skaletsky HJ (2000) Primer3 on the WWW for general users and for biologist programmers. In: Krawetz S, Misener S, editors. Bioinformatics Methods and Protocols: Methods in Molecular Biology. Totowa, NJ: Humana Press. 365–386.
54. Gouy M, Guindon S, Gascuel O (2010) SeaView version 4: A multiplatform graphical user interface for sequence alignment and phylogenetic tree building. Mol Biol Evol 27: 221–224.
55. Tamura K, Peterson D, Peterson N, Stecher G, Nei M, et al. (2011) MEGA5: molecular evolutionary genetics analysis using maximum likelihood, evolutionary distance, and maximum parsimony methods. Mol Biol Evol 28: 2731–2739.
56. Wu X, Zhang C, Feng S, Liu C, Li Y, et al. (2009) Detection of HPV types and neutralizing antibodies in Gansu province, China. J Med Virol 81: 693–702.
57. Kocjan BJ, Gale N, Hocevar Boltezar I, Seme K, Fujs Komlos K, et al. (2013) Identical Human Papillomavirus (HPV) Genomic Variants Persist in Recurrent Respiratory Papillomatosis for up to 22 Years. J Infect Dis 207: 583–587.
58. Ure AE, Forslund O (2012) Lack of methylation in the upstream region of human papillomavirus type 6 from aerodigestive tract papillomas. J Virol 86: 13790–13794.
59. Reidy PM, Dedo HH, Rabah R, Field JB, Mathog RH, et al. (2004) Integration of human papillomavirus type 11 in recurrent respiratory papilloma-associated cancer. Laryngoscope 114: 1906–1909.
60. Rabah R, Lancaster WD, Thomas R, Gregoire L (2001) Human papillomavirus-11-associated recurrent respiratory papillomatosis is more aggressive than human papillomavirus-6-associated disease. Pediatr Dev Pathol 4: 68–72.
61. Brotherton JM, Fridman M, May CL, Chappell G, Saville AM, et al. (2011) Early effect of the HPV vaccination programme on cervical abnormalities in Victoria, Australia: an ecological study. Lancet 377: 2085–2092.
62. Gertig DM, Brotherton JM, Saville M (2011) Measuring human papillomavirus (HPV) vaccination coverage and the role of the National HPV Vaccination Program Register, Australia. Sex Health 8: 171–178.
63. Fairley CK, Hocking JS, Gurrin LC, Chen MY, Donovan B, et al. (2009) Rapid decline in presentations of genital warts after the implementation of a national quadrivalent human papillomavirus vaccination programme for young women. Sex Transm Infect 85: 499–502.
64. Read TR, Hocking JS, Chen MY, Donovan B, Bradshaw CS, et al. (2011) The near disappearance of genital warts in young women 4 years after commencing a national human papillomavirus (HPV) vaccination programme. Sex Transm Infect 87: 544–547.
65. Donovan B, Franklin N, Guy R, Grulich AE, Regan DG, et al. Quadrivalent human papillomavirus vaccination and trends in genital warts in Australia: analysis of national sentinel surveillance data. Lancet Infect Dis 11: 39–44.
66. Kimura M (1980) A simple method for estimating evolutionary rates of base substitutions through comparative studies of nucleotide sequences. J Mol Evol 16: 111–120.
